# All‐on‐4 Implant Supported Prosthetic Rehabilitation of Maxilla After Complete‐Arch Guided Soft Tissue Healing: A 5‐Year Follow‐Up

**DOI:** 10.1155/crid/8816863

**Published:** 2026-01-07

**Authors:** Nazmiye Şen, Pınar Turkoglu, Fırat Selvi

**Affiliations:** ^1^ Department of Prosthodontics, Faculty of Dentistry, University of Istanbul, Istanbul, Türkiye, istanbul.edu.tr; ^2^ Department of Prosthodontics, Faculty of Dentistry, University of Aydın, Istanbul, Türkiye; ^3^ Department of Oral and Maxillofacial Surgery, Faculty of Dentistry, University of Istanbul, Istanbul, Türkiye, istanbul.edu.tr

**Keywords:** all-on-four concept, edentulism, guided soft tissue healing, immediate loading

## Abstract

This case report of a 67‐year‐old patient shows reconstruction of the edentulous maxilla with all‐on‐four implant treatment involving soft tissue esthetics. Reconstruction of the edentulous maxilla involving soft tissue esthetics is a challenging clinical procedure and should be treated with an interdisciplinary approach. In this case report, complete‐arch guided soft tissue healing with an immediate interim prosthesis was applied following all‐on‐four implant placement. The immediate interim prosthesis helped achieve rapid prosthetic rehabilitation and guided soft tissue healing, resulting in an improved esthetic outcome, patient satisfaction, and acceptance for the treatment. Effective communication between surgeons, prosthodontists, and dental technicians made the process easy and successful during both implant placement and prosthetic rehabilitation.

## 1. Introduction

Implant‐supported full‐arch fixed dental prostheses have been used as alternatives to total prostheses in edentulous patients with improved patient satisfaction [1–3]. The required number of implants to support complete‐arch fixed dental prostheses varied among studies from three to eight implants [2, 4–6]. In 2003, the all‐on‐four treatment concept was introduced by Malo et al. to support full‐arch fixed dental prostheses with four implants [1]. It has been considered to be a reliable and effective treatment approach by many other studies then [5–8]. The implant and prosthesis survival rates of the all‐on‐four concept were found to be comparable with those of having more than four implants [7, 8]. Furthermore, the all‐on‐four concept has many advantages such as increasing bone anchorage, decreasing cantilever length, requiring fewer implants, providing immediate function, and reducing the risk of damaging anatomical structures with angled implant placement [2–4, 7, 8]. However, requiring detailed surgical and prosthetic planning with a delicate surgical technique and keeping the cantilever length within limits have been considered the most challenging parts of the concept [2, 9].

The all‐on‐four treatment concept is used to rehabilitate completely edentulous jaws with a fixed dental prosthesis supported by four implants [1–3]. Two implants are placed axially in the anterior region, and two implants are placed distally in the posterior region to reduce the cantilever length [2]. During the surgical phase, a classical flap procedure could be performed with the help of surgical guides prepared using computer‐aided design [10]. Recently, flapless surgery is also possible with the help of individualized guide plates [11]. The use of various guide plates during the surgical phase facilitates the placement of implants at the correct angle and position [10, 12]. Immediate loading can be applied within 2–8 h after implant placement [12–14]. Achieving primary stability is critical for the success of osseointegration [14, 15]. Moreover, immediate loading by splinting implants with full‐arch fixed restorations is reported to have a positive effect on implant survival by accelerating bone healing [15]. Acrylic‐based fixed temporary prostheses without cantilever or with a minimum length of cantilever could be used for immediate loading [6, 14]. The patient who is rehabilitated with the temporary prosthesis should be called for follow‐ups after 1 week, 3 weeks, and 3 months [12–15]. It has been recommended that the construction of permanent prostheses should be started after the temporary prostheses have been used for 4–6 months [8]. Permanent prostheses can be fabricated using metal‐supported ceramic, zirconia, and PEEK material [16–18]. In the present study, a prosthetically guided all‐on‐four implant placement and complete‐arch guided soft tissue healing were applied for the rehabilitation of the maxillary arch with a screw‐retained, monolithic zirconia implant‐supported prosthesis.

## 2. Clinical Report

**Figure 1:**
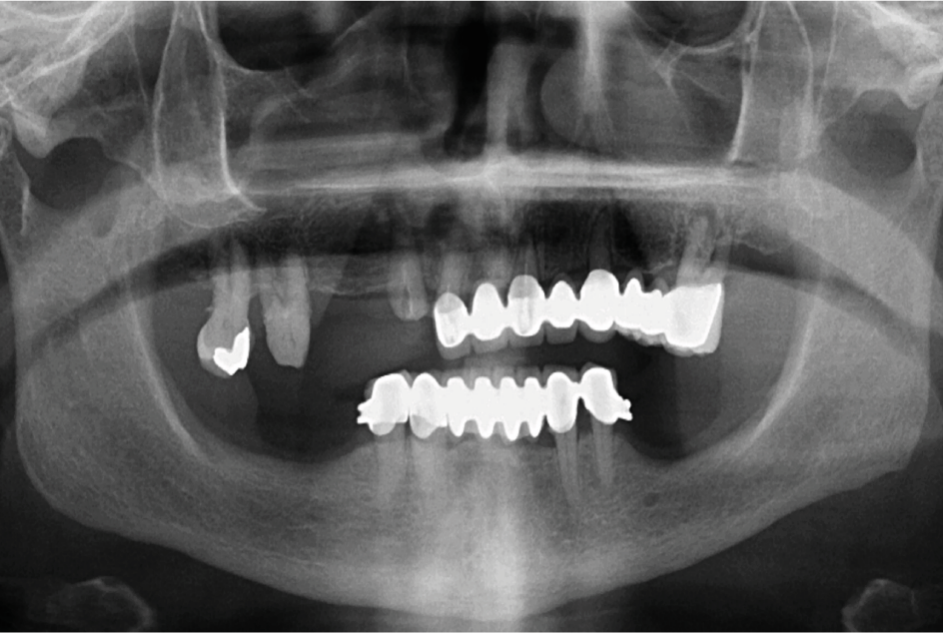
Pretreatment panoramic radiograph.

A 67‐year‐old woman sought care at the Department of Prosthodontics, School of Dentistry, University of Istanbul clinic with a chief complaint of missing teeth in the maxillary arch and with a request for her teeth to be fixed. The patient allowed personal data processing and informed consent was obtained. A panoramic radiograph revealed generalized minor bone loss, missing teeth, and multiple teeth with mobility confirming a terminal dentition status in the maxilla (Figure 1). The patient had a precision attachment denture, and an 8‐unit metal ceramic fixed partial prosthesis in the mandible.

A treatment plan was developed based on clinical and radiographic evaluation to extract all the remaining maxillary teeth. This was to be followed by the placement of four implants in the canine and first molar regions. A monolithic zirconia complete‐arch fixed implant‐supported prosthesis was the selected prosthetic treatment plan for the maxillary arch.

**Figure 2:**
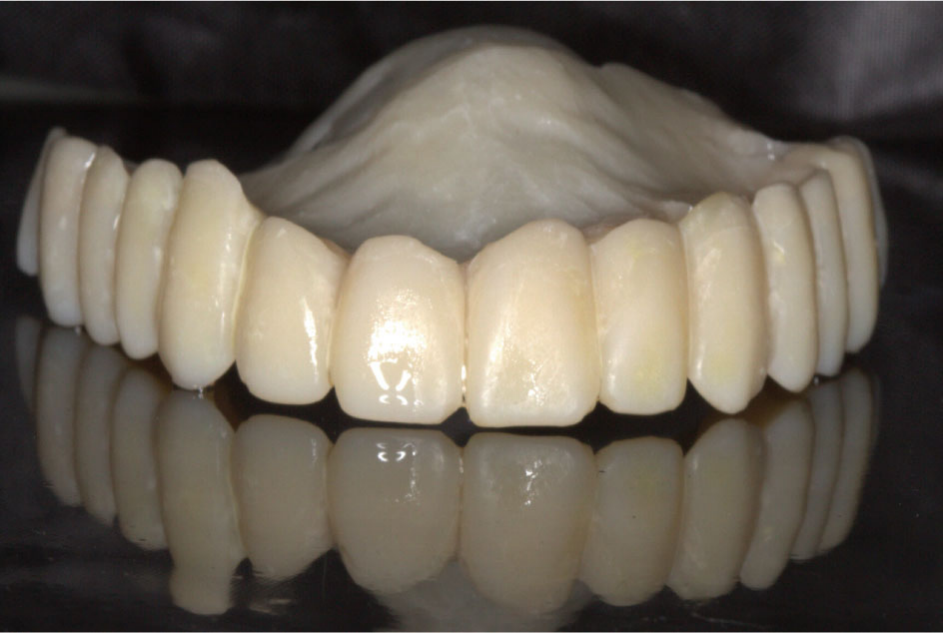
Computer‐aided design/computer‐aided manufacturing prosthetic guide.

**Figure 3:**
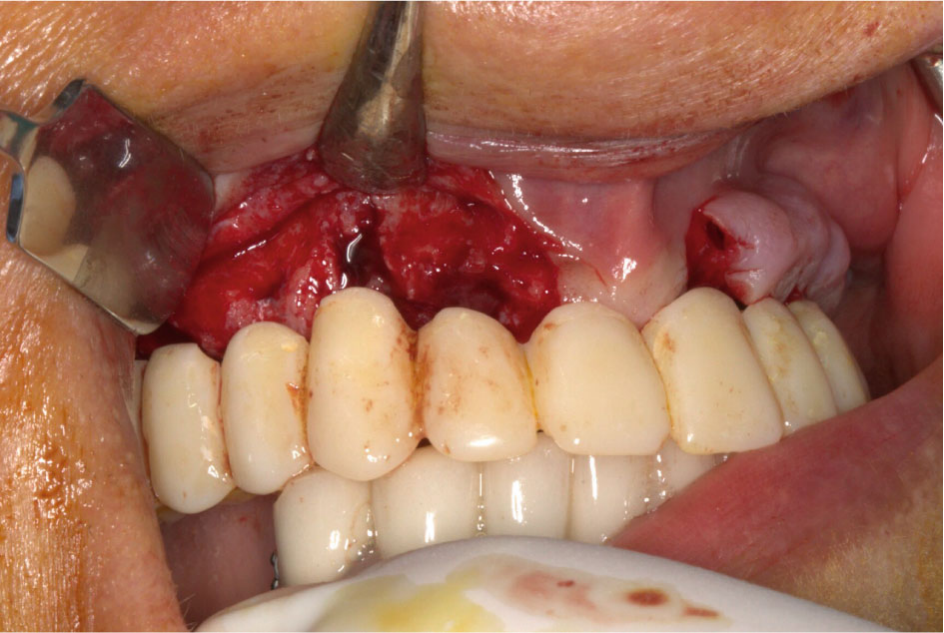
Computer‐aided design/computer‐aided manufacturing prosthetic guide, intraorally fixed for optimized and preplanned implant placement.

**Figure 5:**
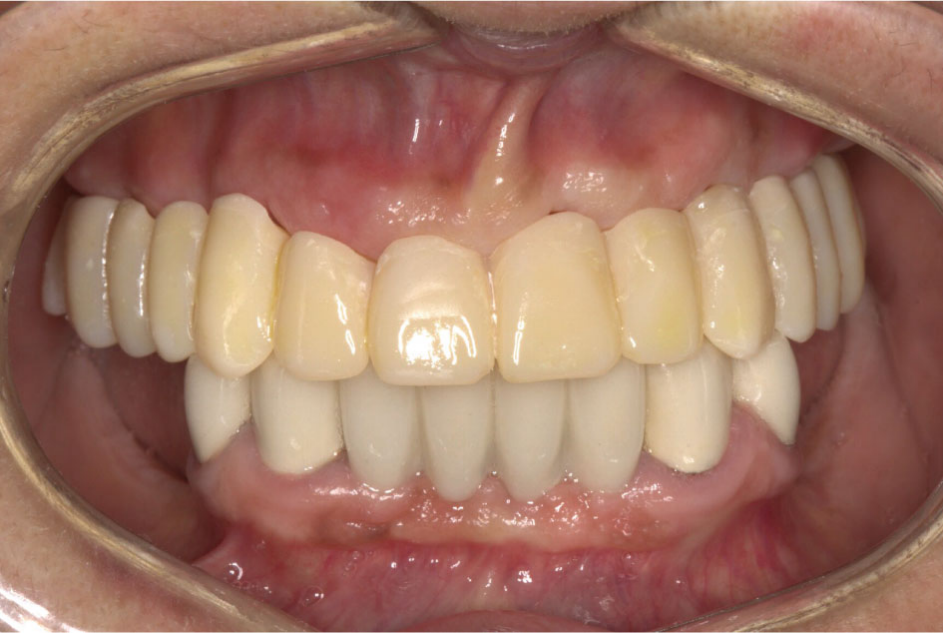
Interim prosthesis.

**Figure 6:**
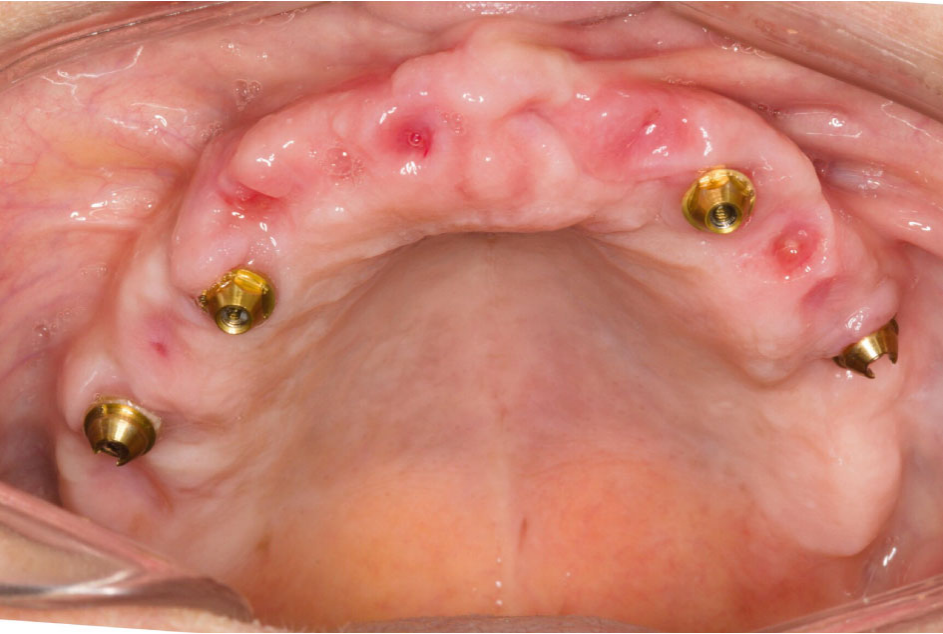
Prosthetically guided soft tissue architecture.

**Figure 8:**
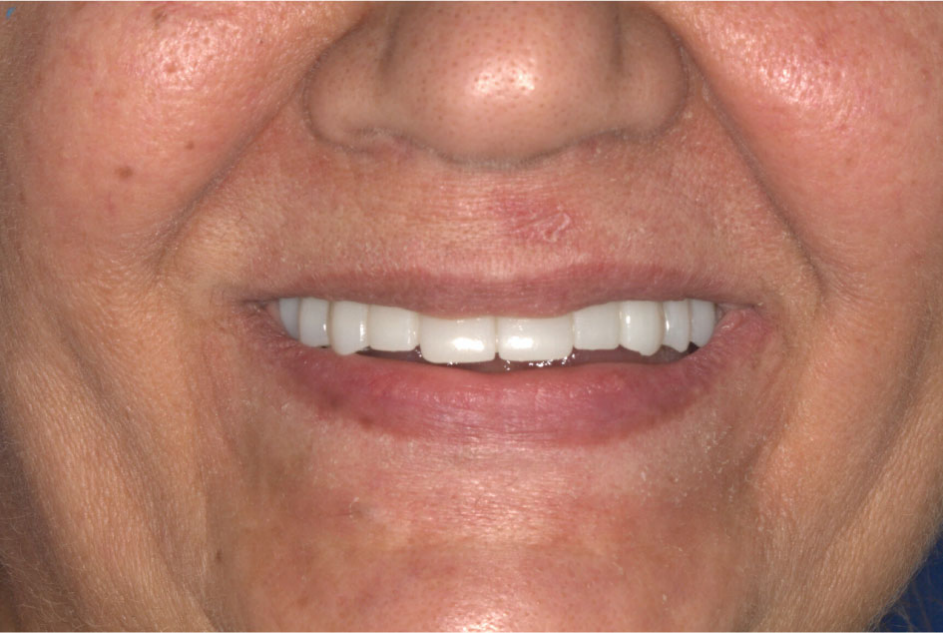
Smile view of definitive prosthesis.

Prior to surgery, diagnostic casts were obtained and mounted on a semiadjustable articulator. An acrylic resin template of the maxillary teeth was prepared, and the vertical dimension of occlusion (VDO) was measured. An immediate maxillary interim prosthesis was then fabricated to be used as a prosthetic guide during implant placement (Figure 2). All the maxillary teeth were removed, and a supracrestal full‐thickness flap was raised following standardized sterile surgical protocols (Figure 3). Four implants were placed in the canine (4.0‐mm Implantium FX 34 10 MLC; Dentium) and first molar regions (4.5‐mm Implantium FX 43 12 MLC; Dentium) (Figure 4). Primary stability was clinically achieved for all implants with an insertion torque greater than 20 N/cm. Therefore, immediate loading was decided to be applied for these implants. A complete‐arch screw‐retained interim prosthesis was placed maintaining the previously determined VDO (Figure 5). A complete‐arch guided soft tissue healing was carried out to create ovate pontics and an ideal emergence profile for the final restorations (Figure 6). Following the placement of the screw‐retained interim prosthesis, posttreatment recalls were scheduled at 2 weeks for the first month and then monthly. A new soft tissue contour was obtained after a 4‐month follow‐up period. No surgical or prosthetic complications were encountered during the osseointegration period. An open‐tray implant impression was made using rigid splinting and polyether impression material (Impregum Penta Soft; 3M ESPE). In the dental laboratory, a maxillary complete‐arch screw‐retained prosthesis was virtually designed and milled using a presintered zirconia block (Prettau; Zirkonzahn) (Figure 7). The zirconia prosthesis was cemented to corresponding titanium cylinders for each implant using a dual polymerizing resin cement (PANAVIA Cement Plus; Kuraray). The zirconia prosthesis was inserted over the four implants, and the passive fit was checked. All the prosthetic screws were then tightened to 15 N/cm following the manufacturer′s instructions. The screw access channels were covered with Teflon tape and light polymerizing composite resin. Follow‐up appointments were scheduled for 6 months at the first year and then annually. Five years after the treatment, no surgical or prosthetic complications were observed (Figure 8).

Figure 4(a) Intraoperative view after implant placement. (b) Postoperative panoramic radiograph.

**Figure:**
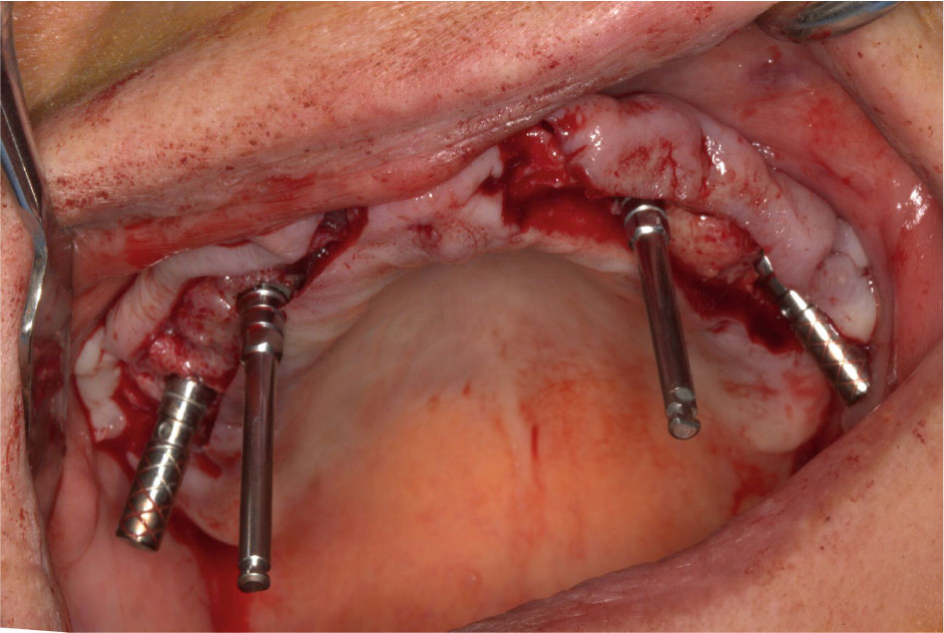
(a)

**Figure:**
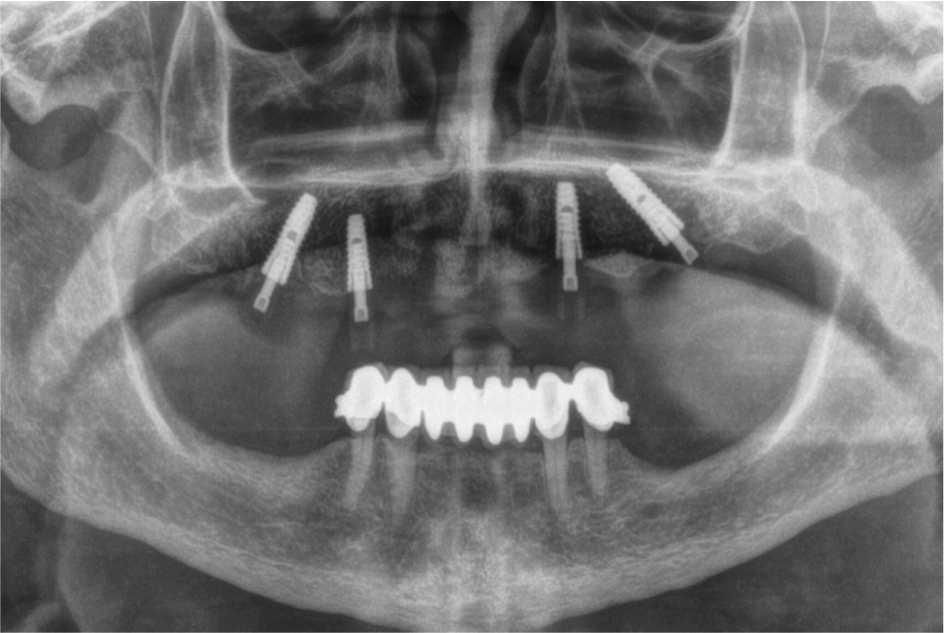
(b)

Figure 7(a) Virtual design of definitive fixed prosthesis. (b) Finished monolithic zirconia prosthesis.

**Figure:**
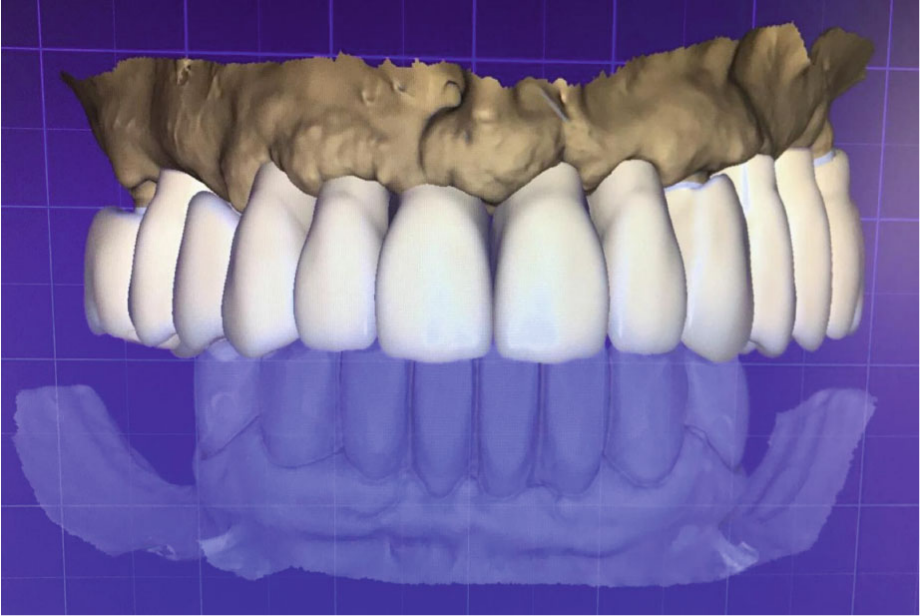
(a)

**Figure:**
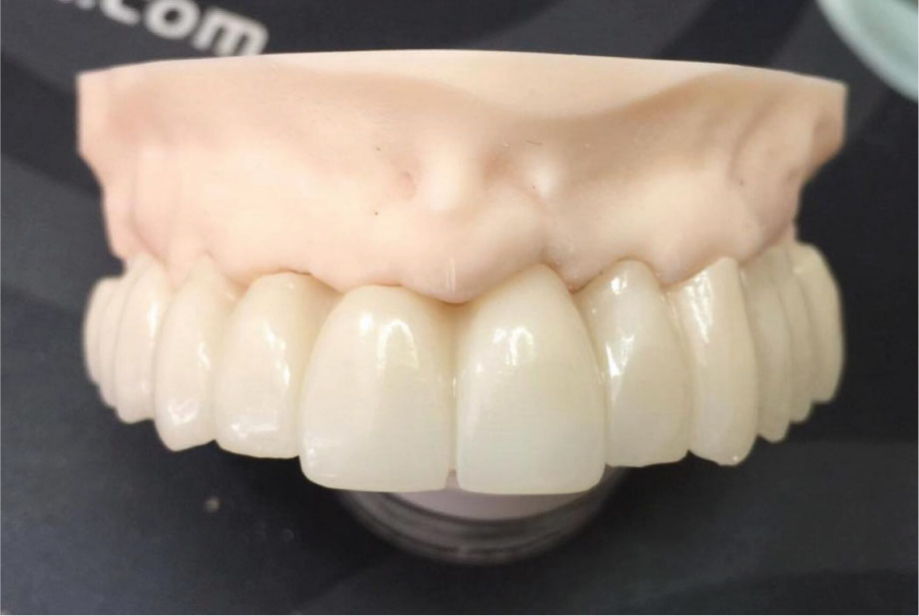
(b)

## 3. Discussion

Reconstruction of edentulous maxilla involving soft tissue esthetics is a challenging clinical procedure and should be treated with an interdisciplinary approach [19, 20]. In this case report, complete‐arch guided soft tissue healing was achieved with the help of an immediate interim prosthesis which also helped to facilitate rapid prosthetic rehabilitation of the patient with improved esthetic outcome, patient satisfaction, and acceptance for the treatment. Effective communication between surgeons, prosthodontists, and dental technicians made the process easy and successful during both implant placement and prosthetic rehabilitation.

Immediate loading with interim restorations is becoming increasingly popular as it expedites treatment and provides rapid prosthetic rehabilitation [13–15]. Immediate loading can be applied when the required primary stability is achieved and there are no signs of pathology [14, 15]. A recent study reported improved patient satisfaction with a high quality of life when immediate loading was applied [14]. In another study that compared clinical outcomes between immediate and delayed loading of dental implants, similar survival rates were reported for both loading modalities [15]. Moreover, it has been stated that patients were satisfied with the results of the treatment in terms of functional, esthetic, phonetic, and psychological aspects [15].

All‐on‐four treatment concept with fixed prosthetic rehabilitation has been considered a reliable treatment modality with high patient satisfaction and survival rates [6, 8, 12]. However, the success rates obtained in studies varied depending on several factors such as follow‐up periods, implant locations, brands, and types of implants placed [1–5, 8, 12]. A cumulative implant survival rate of 96.6% for a 5‐year follow‐up was reported by Lopes et al. [6]. In another study, Malo et al. reported an implant survival rate of 94.8% in the mandible with a prosthesis survival rate of 99.2% for a 10‐year follow‐up [5]. In a 5‐year follow‐up study, the authors reported an implant survival rate of 93% in the maxilla with a prosthesis survival rate of 100% for patients they rehabilitated using different brands and types of implants [21]. The authors concluded the all‐on‐four concept as a viable alternative in the rehabilitation of edentulous patients with high patient satisfaction, reduced cost, and immediate loading advantages [5, 21].

The reestablishment of soft tissue architecture for implant‐supported restorations is fundamental to achieving an esthetic outcome and long‐term implant success [10, 20]. However, it is considered to be challenging to maintain crestal bone levels and preserve gingival form from flattening during the postoperative period [10]. Various techniques including soft and hard tissue augmentation, interim prosthesis with ovate pontics, and subcrestal implant placement have been proposed to mitigate these challenges to create an esthetic outcome with an optimal emergence profile [10–13, 15, 18, 20, 21]. The successful esthetic outcome obtained in this clinical report can be attributed to the immediate interim prosthesis used for both guidance of implant placement and soft tissue healing. Furthermore, the overall esthetic outcome is not only dependent on soft tissue esthetics but also the choice of restorative material plays an important role in achieving an esthetic outcome [16–18]. Several material alternatives are available for the fabrication of definitive prostheses [17, 18]. Metal–ceramic restorations have been frequently used for many years [17]. However, ceramic materials such as zirconia ceramics have been the materials of choice in several clinical situations with their optimal biological, mechanical, and esthetic properties [16–18]. In addition, monolithic zirconia restorations avoid complications commonly encountered with metal–ceramic restorations, such as chipping of the veneering ceramic [17].

## 4. Conclusions

Rehabilitation of edentulous maxilla involving soft tissue esthetics can be achieved with an interdisciplinary approach. Complete‐arch guided soft tissue healing with immediate interim prosthesis following all‐on‐four implant placement helped to achieve satisfactory results with 5 years of follow‐up.

## Conflicts of Interest

The authors declare no conflicts of interest.

## Funding

No funding was received for this manuscript.

## Data Availability

Data is available upon reasonable request.
